# A trial model for medical subspecialty training in South Africa

**Published:** 2016-03-28

**Authors:** P DALMEYER, M STRUWIG, T KRUGER

**Affiliations:** Dept. of Business Management, Nelson Mandela Metropolitan University, Port Elizabeth South Africa.; Dept. Obstetrics & Gynaecology, University of Stellenbosch, South Africa.

**Keywords:** Gynaecology, obstetrics, South Africa, subspecialist training, training model, trial programme

## Abstract

This article outlines the trial model in reproductive medicine that was created as a first step in the development of a business model for medical subspecialty training to complement the current academic subspecialty training in South Africa. A two-tiered training model was developed over time. The hurdles that had to be overcome were the development of a curriculum and academic capacity, acquisition of appropriate funding, acceptance and accreditation of the decentralised training facility, and lastly, registration of the fellowship with the Health Professions Council of South Africa. The end result of the trial programme was a two-year full-time training with supportive funding, or a four-year programme, where the subspecialists would spend three weeks of the month in their home practice environment, attached to an accredited unit, and the last week in an academic institution. Due to the trial program’s success for the South African context and the potential of such model for the developing world, it was evident that the trial programme had to be tested to determine whether and how it can be implemented on a wider basis.

The shortage of healthcare workers and doctors in the developing world compared to the developed world is a problem, and will continue to be so due to the continual migration of qualified professionals and the inability of the state to remedy these shortfalls (Naicker et al., 2009). The demand for subspecialists in reproductive medicine, a subspecialty of obstetrics and gynaecology (O&G), has increased exponentially since the birth of Louise Brown, the first in vitro-fertilised baby in 1978 (Freed et al., 2009). Traditional training curricula for registrars to complete a fellowship in O&G fall well short of what is required for fellowship programmes. This is the reason for the adapted training programme to suite a distant learning programme. This is the case not only in South Africa, but worldwide. Evidence of this is the numerous ad hoc training programmes developed by institutions, to serve this purpose. Programmes have been developed not only for reproductive medicine, but for all subspecialties (Coppus et al., 2007). A review of the literature on subspecialty training in South Africa has yielded minimal to no scientific information. Table I outlines a summary of the research conducted on subspecialty training in South Africa.

**Table I:** — Summary of research on subspecialty training in South Africa.

Author	Main Contribution
Benatar (1997)	Presents a realistic view of reform in South Africa’s healthcare system since the country’s transition to democracy in 1994. Tertiary services and academic training were predominantly offered by government-financed academic hospitals. A concern is the health authorities’ change of focus to primary health care. He proposes a more equitable system of training, where each institution has a different focus.
Benatar (2004)	His concern of the untouched lucrative private medical sector on Johannesburg stock exchange, with its ever-escalating costs, has made little or no effort to contribute to the public sector improving the training environment.
Mullan (2005)	Data indicates that South Africa’s contribution to the total physician workforce of BRICS countries and the world as a whole is substantial. The current trend of migrating physicians will cost South Africa dearly in terms of financial resources (investment in education) and human capital (gifted, ambitious people). The main reason for trainees migrating to developed countries is to achieve personal academic and financial aspirations that cannot be achieved in the source country.
Ogbu (2006)	Support the assertion that source countries should create ethical and effective solutions to counter the worrying trends of human capital migration.
Van Niekerk (2009)	Refers to debates on the duration of medical education that date back to the beginning of the twentieth century. The main concern is the financial burden of training and the future earning capacity of the aspiring specialist and subspecialist, makes the career of specialist and subspecialist financially unsustainable.
Mulder et al. (2010)	They acknowledge the effect of the shortage subspecialists and difficulties inherent in upgrading the existing deteriorating training facilities, the need for new training institutions, and the need to decrease and possibly rationalise the duration of training.
Baleta (2011)	South Africa has an unacceptably high infant mortality rate due to various reasons mainly around skilled workforce
Life Healthcare (2012)	The Life College of Learning which was established by the group in 1998, is provisionally accredited by both the Department of Basic Education and the Department of Higher Education and Training, and by the South African Nursing Council However, this practice has unfortunately not spilled over to the training of specialists in government policy.

Research as indicated in Table I support the contention that a review of subspecialty training, and a transformation of such training, is desperately needed.

Given this background, in order to develop a business model for medical subspecialty training to complement the current academic subspecialty training in South Africa, a trial model for training subspecialists in reproductive medicine was first developed. The trial model in reproductive medicine was essential. It was introduced after it became evident that over the short and medium terms there were not sufficient trained subspecialists qualifying from the academic institutions to fill the voids left by retiring subspecialists. This trial model complemented the current state controlled model of training subspecialists in South Africa as outlined in Figure 1.

**Fig. 1:**
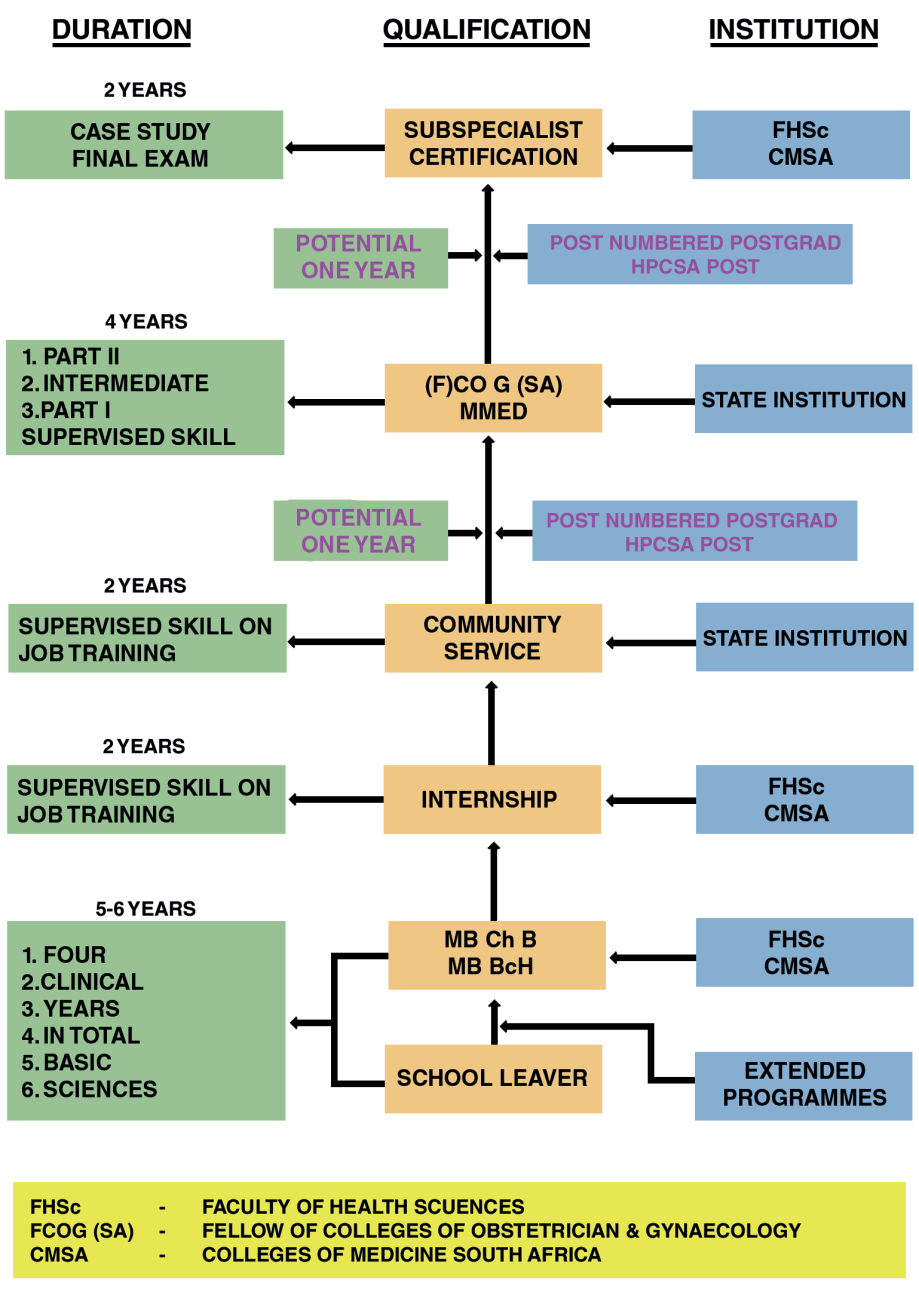
— Current model of training subspecialists in South Africa Source: Authors own compilation.

A two-tiered training model was developed over time. The hurdles that had to be overcome were the development of a curriculum and academic capacity, acquisition of appropriate funding, acceptance and accreditation of the decentralised training facility, and, lastly, registration of the fellowship with the Health Professions Council of South Africa (HPCSA).

The academic institutions developed the curriculum for reproductive medicine as a sub-specialty, it was accepted by the CMSA, and it was registered by the HPCSA in 2005 as a "certification" of this subspecialty. Academic capacity was a challenge in terms of the academic institutions and the provincial authorities, and it continues to be one of the main limiting factors. Funding required lateral thinking, as it would entail financially supporting a full-time fellowship subspecialty student (at a registrar salary scale) for a period of two years. This involved seed funding from the private sector, negotiated with a private hospital group, and further supplemented by donation on a per-treatment-cycle basis (assisted reproductive technology (ART) cycles) from two fertility units.

Accreditation of decentralised training facilities became the responsibility of the Departments of Health Sciences of the medical schools with the help, of the Southern African Society of Reproductive Medicine and Gynaecological Endoscopy (SASREG), and, thus far, 12 units in South Africa have been accredited. Furthermore, the HPCSA has accredited and registered 26 subspecialists in reproductive medicine, under a grandfather clause (capped in December 2010), assessed on the merits of the subspecialists’ curricula vitae and recommendation by peers who are working in the field of the subspecialty, in both public- and private- accredited decentralised units.

It became clear that full-time training capacity would not address future needs. Hence it was decided to investigate decentralised training and this was subsequently implemented on a ‘trial’ scale. Decentralised training provided an opportunity to enrol among O&G specialists in full-time private practice, trainee subspecialists who had an interest in the discipline and a desire to subspecialise. The end result of the trial programme was a two-year full-time training, with supportive funding, or a four-year programme, where the subspecialists would spend three weeks of the month in their home practice environment, attached to an accredited unit, and the last week in an academic institution (hence the two "tiers" of this training programme). From 2002 to early 2015, eight subspecialists have completed fellowships in this manner (Kruger, 2002-2012).

Five of the subspecialists have completed the training programme of two years as full-time trainees. Three of them have completed the programme as decentralised trainees, and two of them are currently enrolled as decentralised trainees. The funding for these trainees has been generated from self-funding, as well as from funding raised from the private sector, namely from hospital groups.

Inadequacies in the training programme, such as in endocrinology, menopausal medicine and endoscopic surgery are handled by two academic institutions that are involved, to ensure that the training fellow will cover all aspects of the curriculum. In 2015 a third unit has managed to train two subspecialists, through a registration programme for private subspecialists on the institution’s academic staff. A fourth unit is under consideration, but will be run by four private subspecialists part-time employed by an academic institution. The viability of this model is not clear. The training of these two subspecialists has been unpaid, and they have expressed a desire to participate in distance training, if allowed to do so.

The public and the private sector are increasingly experiencing shortages of both specialists and subspecialists. The trial model that has been developed is the first attempt to address these threatening shortages. Such a model can be applied to other subspecialties with similar shortages. The three major hospital groups in South Africa as well as other educational trusts and the medical business sector have over the years taken the initiative to fund education projects in conjunction with the country’s academic institutions. However, these projects have been poorly focused and have been managed in an uncoordinated way. It is clear that there is a desire on the part of the hospital groups and other large companies via foundations to become involved in these projects on a much larger scale. Medical insurers have become major sponsors of fellowship trust programmes in specialist and subspecialist training, but the funding has usually been done ad hoc and unstructured.

Due to the trial programme’s success, for the South African context and the potential of such model for the developing world, it was evident that the trial programme had to be tested to determine whether and how it can be implemented on a wider basis. This was done as part of a PhD study at the Nelson Mandela Metropolitan University of South Africa (Dalmeyer, 2015).
